# Thermography in Stroke—A Systematic Review

**DOI:** 10.3390/medicina61050854

**Published:** 2025-05-06

**Authors:** Anna Podlasek, Ivo Petrov, Zoran Stankov, Kenneth Snyder, Carlos Alejandro Alvarez, Piotr Musialek, Iris Q. Grunwald

**Affiliations:** 1 Image Guided Therapy and Research Facility (IGTRF), University of Dundee, Dundee DD1 4HN, UK; 2 Nottingham Biomedical Research Centre (BRC), University of Nottingham, Nottingham NG7 2RD, UK; 3 Cardiology and Angiology Department, Acibadem City Clinic Cardiovascular Center, 1407 Sofia, Bulgaria; 4 Neurosurgery, University at Buffalo, Buffalo, NY 14068, USA; 5 Hospital Privado Del Sur, Las Heras, Bahia Blanca 8000, Argentina; 6 Department of Cardiac and Vascular Diseases, John Paul II Hospital, Jagiellonian University, 31-007 Krakow, Poland

**Keywords:** stroke, thermography, triage, systematic review

## Abstract

*Background and Objectives*: Thermography is a non-invasive diagnostic technique that measures skin surface temperatures to reflect normal or abnormal physiology. This review explores the clinical utility of thermography in diagnosing and monitoring stroke, with an emphasis on its clinical applications. *Materials and Methods*: This systematic review followed PRISMA guidelines, with a protocol published prior to analysis. Three databases were screened up to end of 2024. Article selection was conducted in two stages: title and abstract screening using Rayyan^®^, followed by full-text eligibility assessment. Discrepancies were resolved through consensus. Risk of bias assessment was performed with ROBINS-I. Narrative synthesis was planned in addition to descriptive statistics. *Results*: A total of 20 studies were included after screening 277 records. Thermography emerged as a promising tool for stroke patients in both the acute and chronic phases. In the acute phase, it demonstrated potential in detecting early signs of carotid occlusive disease by identifying temperature differences in the forehead or neck regions. Additionally, thermography contributed to the differential diagnosis of Wallenberg syndrome. In the chronic phase, it exhibited clinical utility in monitoring rehabilitation progress. *Conclusions*: Thermography shows promise as a non-invasive tool for stroke assessment and monitoring. While preliminary studies suggest physiological relevance, its clinical utility remains investigational and requires further validation.

## 1. Introduction

Thermography is a non-invasive diagnostic technique that measures skin surface temperatures to reflect normal or abnormal physiology [1]. It is based on the principle that the human body emits infrared radiation, which can be detected and translated into thermal images. This makes thermography a safe and painless procedure without any exposure to radiation [2]. Thermal asymmetry, a difference in temperature between corresponding body parts, is often associated with underlying physiological or pathological processes [3], and difference a greater than 0.5 °C is often considered as abnormality [4,5].

In the context of cerebrovascular disorders such as ischemic stroke, thermography can be used to assess the state of the autonomic nervous system, which is often affected in these conditions [6,7,8]. Sympathetic dysfunction, a hallmark of cerebrovascular disorders, can manifest as temperature variations on the skin. For example, in cases of ischemic stroke, skin temperature asymmetry has been noted, particularly in the presence of pyramidal tract signs or Wallenberg’s syndrome [9].

**Figure 1:**
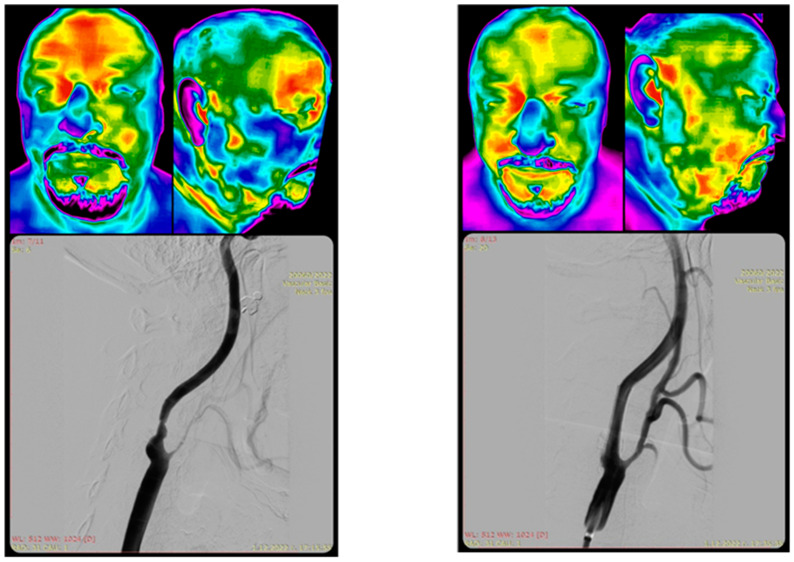
Thermographic imaging before and after carotid stenting in a 62 yo patient with significant right internal carotid artery (RICA) stenosis and right external carotid artery (RECA) occlusion. From the kind contribution from IP (patient image).

Thermography has the theoretical potential to be a useful tool in the emergency diagnosis and triage of stroke patients [2]. It can detect early signs of carotid occlusive disease by identifying temperature differences in the forehead or neck region, an area supplied by the internal carotid artery [10]. Reduced blood flow due to compromised arteries can lead to cooling of the skin in the area affected [11] (Figure 1).

The patient had a history of ipsilateral transient ischemic attack (TIA) two months prior to the procedure. Left panel (pre-stenting): Thermogram demonstrates prominent cold (blue/green) coloration over the right maxilla and mandible, consistent with regional hypoperfusion due to RECA stenosis. Additionally, cooler temperatures over the right frontoparietal skull area correspond to hypoperfusion in the middle cerebral artery (MCA) territory, reflecting reduced intracranial flow. Right panel (post-stenting): Thermogram shows rewarming and thermal symmetry restoration in the same regions, with warmer (yellow/red) tones suggesting improved perfusion following carotid revascularization. 

The review aims to evaluate thermography in two main clinical contexts: (1) the acute phase, for early diagnosis of stroke and stroke mimics, or differentiation between stroke subtypes, exploring both diagnostic and prognostic application; (2) the chronic phase, for monitoring of rehabilitation progress and neurological recovery in chronic stroke survivors.

## 2. Materials and Methods

### 2.1. Search Strategy

This systematic review was conducted following the Preferred Reporting Items for Systematic Reviews and Meta-Analyses (PRISMA) guidelines [12], with the protocol published prior to the analysis [13]. We systematically searched electronic databases from inception until 10 October 2024, including PubMed, Scopus, and Cochrane. A follow-up search was performed on 31 December 2024 to ensure that no new evidence was missed. The search phrase for Pubmed was “((((stroke) OR (infarc*)) OR (cva)) OR (cerebrovascular accident*)) AND (thermograph*)”, and was adapted to “(TITLE-ABS-KEY(stroke OR infarct* OR cva OR “cerebrovascular accident*”)) AND (TITLE-ABS-KEY(thermograph*))” for Scopus and “(stroke OR infarct* OR cva OR “cerebrovascular accident*”) AND thermograph*” for Cochrane. There were no limits within search strategy applied.

Although thermography has been applied across various stages of stroke detection and management, this review adopts a systematic approach to evaluate the evidence within well-defined stroke phases: acute and chronic. This structured focus, combined with formal quality assessment of included studies, justifies the use of a systematic review methodology over a scoping review [14]. This approach was previously used for different condition such as rheumatic diseases [15,16], tendinopathy [17], systemic vasosonctriction [18], acute illness [19] or general insight into thermography as a screening and diagnostic tool [20].

### 2.2. Study Selection

Article selection occurred in two stages: initially, two reviewers (AP, IG) independently screened the titles and abstracts for relevant studies using Rayyan^®^ [21]. In the second stage, they independently assessed the full texts for eligibility. Any discrepancies were resolved through discussion and consensus. Additionally, reference lists of the included publications were hand-searched for further relevant studies.

### 2.3. Eligibility Criteria and PICO Framework

Studies were included based on the following criteria, structured around the PICO framework [22]: 

Population (P): Adult patients (≥18 years) with a confirmed diagnosis of stroke (both ischemic and haemorrhagic), including both acute and chronic presentations.

Intervention/Index Test (I): Use of infrared thermography to evaluate thermal asymmetries, monitor physiological changes, or assess rehabilitation outcomes in the context of stroke.

Comparator (C): When applicable, comparisons included contralateral body regions (e.g., affected vs. unaffected limb or hemisphere), healthy controls, or reference imaging modalities (CT, MRI, Doppler ultrasound). However, due to the observational nature of most included studies, the presence of a formal comparator was not required.

Outcomes (O): Key outcomes included identification of thermal asymmetry, feasibility and diagnostic utility of thermography, correlation with clinical or functional status, and responsiveness to therapeutic interventions.

We included peer-reviewed studies of any design (e.g., case reports, observational studies, randomized trials) published in English that investigated thermographic imaging in stroke patients. Exclusion criteria comprised studies involving non-human subjects, stroke-unrelated thermography, conference abstracts, and reviews or meta-analyses.

### 2.4. Data Extraction and Management

The following data were systematically extracted: author, year of publication, country of study, study design, number of participants, thermography system utilized, thermography software employed, pre-imaging instructions, room specifications, body position/region of interest (ROI), visualized body area, stroke phase, intended use of thermography, results, and conclusions.

### 2.5. Statistical Analysis

Study characteristics and extracted variables were summarized using standard descriptive statistics. Categorical variables were expressed as frequencies or percentages. To evaluate the methodological quality of the included studies, we applied study-design-appropriate risk-of-bias tools. For randomized controlled trials, we used the RoB 2 tool, which assesses bias across five domains, including randomization, deviations from intended interventions, missing outcome data, measurement of outcomes, and selection of reported results [23]. Non-randomized interventional studies were assessed using ROBINS-I, which evaluates bias due to confounding, participant selection, classification of interventions, deviations from intended interventions, missing data, outcome measurement, and selection of the reported results [24]. For observational cohort and case-control studies, we used the Newcastle–Ottawa Scale (NOS), a widely recognized tool that assesses studies based on selection, comparability, and outcome [25]. Cross-sectional studies were assessed using either the AXIS tool, designed specifically for appraising cross-sectional designs [26], or the JBI Checklist for Analytical Cross-Sectional Studies, depending on the study’s structure [27]. Case reports were evaluated using the JBI Checklist for Case Reports [27], and the JBI Case-Control Checklist was applied to case-control studies [27]. Studies that reported diagnostic accuracy metrics such as ROC curves were assessed using the QUADAS-2 tool, which evaluates bias related to patient selection, index tests, reference standards, and flow/timing [28]. Each assessment was performed by two reviewers. Disagreements were resolved through discussion and consensus. The overall risk of bias was categorized as low, moderate, or high.

### 2.6. Data Availability Statement

All data used for analyses are available within the original publications of the included studies. The summary dataset is available in the online repository [29].

## 3. Results

**Figure 2:**
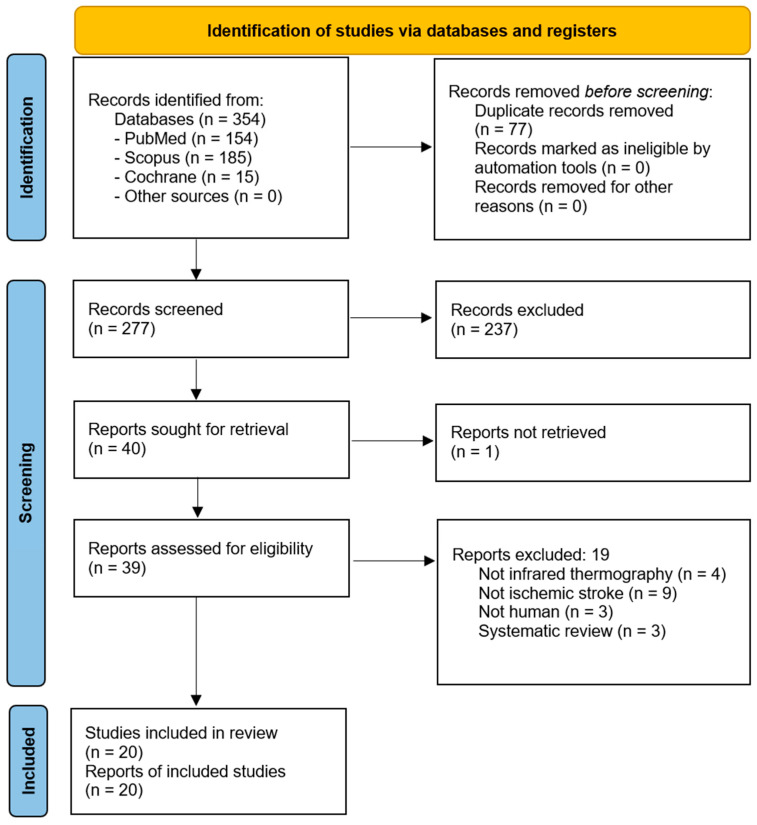
PRISMA flow chart of study screening and selection.

Our search strategy identified 354 studies, which, after title and abstract screening, yielded 39 full-text articles for evaluation. We excluded 19 studies based on the reasons displayed in the PRISMA flow chart (Figure 2), with the details documented in Supplementary Table S1. A total of 20 individual papers met the inclusion criteria and were thus included in our systematic review (Table 1). They reported on 722 participants.

### 3.1. Study Design and Settings

The most common study design across is the cross-sectional study, appearing seven times (35%) [31,35,37,38,39,40,41]; similarly, prospective studies were also featured seven times (35%) [9,32,43,44,45,48,49]. Observational studies were also frequent, appearing five times across both groups (25%) [32,33,34,37,47]. There were four case reports [30,36,37,46].

The settings for the studies varied, encompassing rehabilitation facilities, hospitals, and university departments [1,9,30,31,32,33,34,35,36,37,38,39,40,41,43,44,46,47,48,49,50].

### 3.2. Instructions Prior to Imaging

In preparation for thermographic imaging, several key instructions were frequently emphasized to ensure accurate results. Patients were most often advised to avoid vigorous exercise and the consumption of stimulants, such as alcohol, coffee, or caffeine [35,36,38,39,40,41,44,48,49]. Instructions to avoid hot showers or baths [35,36,38,39,44], and the application of lotions, creams, or powders [35,36,38,41,48] were each highlighted five times, as was the need for a 15–30 min acclimatization period in a temperature-controlled room [9,31,36,45,47]. Additional guidance included refraining from heavy meals (mentioned three times) [35,39,41], removing gloves, socks, shoes, or jewelry [35,40,45], and avoiding scratching the body [36,40,48], each noted three times. Patients were also instructed twice to wear minimal clothing [35,40], avoid nasal decongestants [39,40], and refrain from pressing or scratching the face. Other less-frequently mentioned instructions included avoiding physical treatments before the examination [44] and undergoing specific imaging protocols involving ice water recovery [47].

These instructions were intended to standardize conditions and ensure reliable thermographic measurements by reducing the impact of external factors on skin temperature. The specific duration for abstinence from these activities or substances varied, but a two-hour window was frequently specified before the thermographic assessments [36,39,40].

### 3.3. Room Settings

The room settings for thermographic imaging consistently emphasized maintaining a controlled environment to ensure accurate results [1,9,30,31,32,33,34,35,36,37,38,39,40,41,43,44,46,47,48,49,50]. The most commonly reported temperature range was between 21 °C and 24 °C, mentioned multiple times [38,39,44], with some studies specifying an average room temperature of 22 °C [44]. Humidity levels were typically kept between 40% and 65% [39,44], with a few studies noting higher humidity levels of up to 80% [38]. Ensuring that the room was draft-free was a repeated requirement, with windows, doors, and curtains often closed to maintain a stable environment. Additionally, lighting was described as cold fluorescent, and efforts were made to reduce external light. The absence of air conditioning or radiators was noted to avoid temperature fluctuations, supporting a consistent ambient setting for the imaging process (Supplementary Table S2).

These controlled room conditions were intended to ensure that any observed temperature differences were due to physiological factors, rather than variations in the external environment. This standardization is crucial for the reliability and validity of thermographic assessments in research settings. Some studies acknowledge that these conditions were not always possible to regulate.

### 3.4. Visualized Body Parts

The whole body was visualized six times, making it one of the most frequently examined areas [9,33,34,38,40,43]; however, it was often composed of smaller parts combined [33,34,40] rather than a projection of the full body [9,38,43]. Visualization of the upper and lower limbs was reported four times [30,35,39,48], matching the frequency of lower-limb visualization alone [36,37,44,45]. Upper limbs were specifically visualized three times [46,47,49]. The head was visualized in one study [31], while the face appeared in two studies [32,41]. Additionally, the feet were visualized two times [35,39], with the plantar region of both feet being specifically mentioned in one study [37] (Table 1).

### 3.5. Intended Use

Thermography has been explored as a useful tool in both acute and chronic stroke management, facilitating diagnosis, monitoring, and rehabilitation [1,9,30,31,32,33,34,35,36,37,38,39,40,41,43,44,46,47,48,49,50]. In the acute phase, it is primarily used to assess skin temperature asymmetries, which can signal autonomic dysfunction, and to monitor body and limb temperature differences to track stroke progression [9,30,31,32,33,34,50]. Several studies reported cooler temperatures on the paretic or hypoperfused side of the body. For instance, Park et al. [30] noted a ~2.0 °C drop in limb temperature opposite to the side of lacunar infarction, and Piskorz et al. [31] observed a mean temperature decrease of 0.49 °C on day 1 and 0.38 °C on day 4 in the infarcted hemisphere compared to the unaffected side. Moreover, Takahashi et al. [33] documented laterality differences (LDs) in body surface temperature between 1.0 °C and 2.5 °C in patients with Wallenberg syndrome, particularly in the abdomen and limbs, absent in control subjects with non-central vertigo.

Studies have also investigated brain temperature variations to assist in differentiating ischemic from hemorrhagic strokes, with early diagnostic accuracy being critical in reducing morbidity [31]. Additionally, thermography of facial temperature has shown potential as a marker for delirium [32], while body surface temperature measurements can aid in diagnosing Wallenberg’s syndrome, distinguishing it from pontine infarction or noncentral vertigo [34,50]. For example, temperature asymmetry in Wallenberg’s syndrome has been found to be distinct compared to other conditions, enhancing diagnostic precision [34,50].

Thermography provides insight into chronic-phase post-stroke recovery and rehabilitation [1,35,36,37,38,39,40,41,43,44,46,47,48,49,50]. It is used to evaluate thermal sensitivity differences in hemiplegic patients compared to healthy controls and to monitor temperature changes during and after therapies such as robotic-assisted gait training. Da Silva Dias et al. [40] reported that patients with stroke sequelae had an average temperature asymmetry of 0.7 °C between the plegic and non-plegic limbs. Alfieri et al. [36] recorded temperature increases after robotic-assisted gait training, which returned to baseline within 30 min, demonstrating thermography’s role in tracking therapy response. Portable thermographic devices have made it easier to detect thermal asymmetry, with studies reporting good agreement with high-resolution cameras. Thermographic analysis also helps quantify limb temperature to assess sensorimotor recovery, track whole-body temperature distribution in patients with side-to-side complaints, and evaluate plantar temperature in those with stroke and diabetes [37]. Furthermore, changes in muscle temperature have been linked to rehabilitation outcomes, such as reduced spasticity, improved microcirculation, and joint function. In stroke survivors with pain disorders, thermographic monitoring of temperature changes has been correlated with pain relief, particularly for central post-stroke pain, where improvements in central post-stroke pain were accompanied by an increase in skin temperature at the previously cooler pain site [43]. Additionally, the technique has been used to study the effects of rehabilitation strategies, such as virtual reality, on body symmetry and functional recovery. For example, monitoring the shank’s surface temperature can reflect spasticity reduction, while temperature variances in jaw muscles or hemiplegic arms provide objective measures of recovery [41,44,48]. These findings underscore the value of thermography as a non-invasive, cost-effective tool to support both diagnosis and therapeutic interventions in stroke care.

### 3.6. Limitations of Thermography

There are inherit limitations of infrared thermography that contributed to the fact that despite the recent progress and clinical research, it has not yet been adopted in the clinical guidelines as a standalone imaging tool [51]. It only measures surface temperature and does not detect deeper ischaemic damage [30,52]. Also, other factors may influence skin temperature [32], so the method lacks specificity [31]. Additionally, while temperature differences are a clear indicator, they should be interpreted alongside other clinical symptoms and tests. Infrared thermography lacks harmonized standards, with variability in equipment, environmental conditions, and the absence of a unified diagnostic framework contributing to inconsistent results [53]. Its accuracy is also affected by subject-specific factors such as sweating, circadian rhythms, body composition, and physiological states, which can obscure or mimic pathological findings [51]. Moreover, thermography has lower precision and diagnostic specificity compared to established imaging modalities like CT or MRI, limiting its role to that of a complementary tool rather than a standalone diagnostic method [51].

### 3.7. Risk of Bias

The included studies in this systematic review encompassed a variety of methodological designs, including case reports, cross-sectional studies, observational cohorts, randomized controlled trials (RCTs), and non-randomized interventional designs. Given this diversity, appropriate risk-of-bias tools were applied to each study type to ensure methodological rigor and accurate appraisal of internal validity.

For cross-sectional studies, the AXIS tool [35,38,39,40,41,45] and JBI checklist for cross-sectional studies were employed. These studies typically demonstrated moderate-to-good methodological quality, though concerns were noted around incomplete reporting, convenience sampling, and lack of blinding. Several studies had moderate risk due to insufficient detail on sampling methodology or participant recruitment.

Case reports [30,36,46] were evaluated using the JBI Checklist for Case Reports, which found generally low risk of bias across all such studies. These were well reported, with clearly defined patient characteristics and diagnostic pathways, though inherent limitations in generalizability were acknowledged.

Case-control [37] and cohort studies [9,32,47] were assessed using the JBI Case-Control Checklist and Newcastle–Ottawa Scale (NOS), respectively. These studies often demonstrated good selection and outcome domains but showed variable comparability due to inconsistent control matching or adjustment for confounders.

One study reported diagnostic accuracy metrics such as ROC curves [31]. For these, the QUADAS-2 tool was applied. These studies were generally of moderate quality, with low risk in patient selection and index test domains, but some uncertainty remained in the application and interpretation of the reference standard.

For non-randomized interventional studies [33,34,48], the ROBINS-I tool was used. While most scored low to moderate across domains such as confounding and selection bias, measurement bias and lack of blinding were recurrent concerns. These affected the certainty with which causal inferences could be drawn from temperature asymmetry changes following interventions.

The sole randomized controlled trial [49] was evaluated using the RoB 2 tool, which revealed moderate risk of bias due to some concerns in the handling of deviations from intended interventions and incomplete outcome data.

While the overall risk of bias across the included studies ranged from low to moderate, no studies were classified as high risk. The most frequent limitations were small sample sizes, lack of blinding, inadequate control groups, and variability in reporting standards. These findings underscore the need for standardized protocols and improved methodological rigor in future thermography studies in stroke care.

The detailed scoring is available in Supplementary Table S3.

## 4. Discussion

This systematic review examined 20 studies involving 722 participants to assess the use of thermography in stroke. The findings suggest that thermography has potential as a non-invasive diagnostic tool for stroke, with applications in diagnosis, monitoring, and potentially in triage. However, the review also highlighted several limitations and potential sources of bias that need to be addressed in future research.

### 4.1. Thermography in Medicine

Thermography has been employed across various medical fields due to its non-invasive nature and ability to detect subtle temperature variations indicative of underlying physiological changes. In oncology, it is used to identify areas of increased metabolic activity associated with tumor growth [54]. In musculoskeletal disorders, thermography aids in detecting inflammation and monitoring treatment responses [17]. In vascular medicine, it is utilized to assess peripheral vascular diseases by identifying regions with compromised blood flow [52]. In neurology, beyond stroke, thermography has applications in diagnosing conditions like complex regional pain syndrome and neuropathies, where it helps in visualizing temperature asymmetries linked to autonomic dysfunction [54,55,56]. In these contexts, thermography is typically used to monitor chronic physiological abnormalities or screen for localized vascular compromise. Compared to its use in cancer or diabetes—where pathological changes are more localized or sustained—stroke-related thermal patterns are often transient, dynamic, and more variable, necessitating careful interpretation and complementary diagnostic modalities [1,10]. Recent advancements in infrared camera technology and data processing techniques have enabled real-time, high-resolution thermographic imaging, opening up new possibilities for research and clinical applications [51,57]. In the context of stroke risk factors, thermography has demonstrated the ability to detect early signs of carotid occlusive disease (carotid stenosis) by identifying temperature differences in the forehead or neck regions, which are supplied by the internal carotid artery [10,58]. This correlation with carotid imaging suggests that thermography could potentially serve as a complementary tool in stroke risk assessment and management.

Lin et al. demonstrated the further potential of infrared thermography as a real-time, non-invasive, contrast-free alternative to indocyanine green videoangiography for assessing anastomosis patency in moyamoya syndrome bypass surgery [59]. Their preliminary study showed perfect agreement between thermography and ICG-VA in detecting anastomotic patency across 21 patients, including one case where both methods identified an obstruction. This application of thermography offers several advantages, such as avoiding contrast agent-related risks and providing real-time, easily interpretable pseudo-color images of blood flow [48], which may provide a good parallel for the urgent need for immediate brain imaging in case of suspected stroke.

### 4.2. Acute Versus Chronic Stroke

Infrared thermography serves distinct clinical and pathophysiological roles in acute versus chronic stroke management. In the acute phase, thermography can detect asymmetrical skin temperature distributions resulting from impaired cerebral perfusion. For instance, studies have demonstrated that infrared thermography can detect temperature differences in facial regions of acute stroke patients, correlating with cerebral perfusion deficits [32]. This non-invasive, rapid assessment tool can aid in early stroke detection, especially in settings lacking immediate access to advanced imaging modalities.

In contrast, during the chronic phase, thermography is valuable for monitoring rehabilitation progress by assessing changes in muscle tone and spasticity. Research has shown that thermographic assessments can reveal microcirculatory dysfunctions in affected limbs of chronic stroke patients, with temperature increases correlating with improvements in joint function following rehabilitation [44].

### 4.3. Thermography Cameras and Systems

The studies reviewed utilized various thermographic systems, each with unique characteristics that impact their clinical utility. For instance, high-resolution cameras like the FLIR T650SC provided detailed thermal imaging advantageous for detecting subtle differences, particularly useful in rehabilitation monitoring [38]. However, lower-resolution systems or portable models, despite good general concordance with higher-end models [38], might fail to detect minimal yet clinically significant thermal asymmetries critical in acute diagnostics or precise monitoring. Portable devices, like FLIR C5, represent promising options for wider implementation due to cost-effectiveness and ease of use, but additional large-scale validation is crucial before broad adoption [38]. The choice of the optimal device should be guided by the specific research question. If widespread adoption is considered, a formal health technology assessment process is recommended [60].

The choice of region of interest (ROI) significantly influences thermography’s diagnostic and prognostic capabilities. While whole-body thermography offers comprehensive insights into systemic effects and autonomic dysregulation associated with stroke, it is currently logistically demanding and clinically uncommon. Most available clinical thermographic devices focus on localized assessments such as limbs or facial regions, capturing critical information for specific stroke subtypes, e.g., Wallenberg syndrome [34,50]. Still, they risk missing relevant thermal changes elsewhere.

### 4.4. Strengths and Limitations of the Included Studies

The studies included in this review offered valuable insights into the clinical applicability of thermography across acute and chronic stroke care. A key strength lies in their real-world settings—many were conducted in rehabilitation centers or general hospitals, reflecting diverse patient populations and clinical scenarios (Table 1 and Table S2). Some studies demonstrated methodological rigor, with prospective designs, detailed thermographic protocols, and well-defined outcomes [49]. Furthermore, the consistency of findings related to thermal asymmetry in hemiparetic limbs or specific syndromes (e.g., Wallenberg) supports the physiological relevance of thermography in stroke contexts (Table 1 and Table S2).

However, several limitations were also evident. Many studies were small in scale, with limited sample sizes that constrain generalizability (Table 1, Tables S2 and S3). Methodological heterogeneity was common, especially in the selection of thermographic equipment, patient preparation protocols, and environmental conditions (Table 1 and Table S2). Few studies included formal comparator groups or blinding, and many lacked follow-up, limiting causal inference (Table 1, Tables S2 and S3).

### 4.5. Comparison with Established Imaging Modalities

Compared to gold-standard diagnostic tools—MRI, CT, and Doppler ultrasound—thermography offers unique advantages and notable limitations. Unlike MRI and CT, which provide anatomical and perfusion-based insights into intracerebral pathology, thermography is limited to surface temperature measurements and lacks spatial resolution to detect deep ischemic changes [61]. It cannot currently differentiate between ischemic and hemorrhagic stroke with high accuracy, nor can it localize infarction [31].

However, thermography offers several pragmatic advantages: it is entirely non-invasive, involves no radiation, and requires minimal patient cooperation. Imaging can be conducted in under two minutes and does not require specialized infrastructure, making it particularly attractive in low-resource settings or as a rapid triage adjunct in prehospital care (Table 1 and Table S2). Portable, battery-operated devices are now available at a fraction of the cost of CT or MRI systems, and some have shown high concordance with clinical-grade thermographic cameras [38].

Furthermore, thermography avoids the use of contrast agents (as used in CT angiography or MRI with gadolinium) and may be repeated multiple times to monitor dynamic changes during stroke evolution or rehabilitation. Considering that it visualizes physiological and functional abnormalities rather than anatomical abnormalities, it can improve monitoring diseases difficult to diagnose with CT or MRI, such as neuropathic pain, headache, and myofascial pain [62]. While its current role is best seen as complementary—e.g., aiding in triage or monitoring rather than diagnosis—it could feasibly integrate into clinical workflows with minimal training, particularly for tracking therapy response or autonomic dysregulation.

Future integration would benefit from harmonized imaging protocols, automated temperature asymmetry detection, and cross-validation with established modalities. Emerging machine learning approaches may also enhance interpretability and facilitate real-time decision support.

### 4.6. Limitations

This systematic review has several important limitations that should be acknowledged. First, although we followed a structured search protocol based on PRISMA guidelines [12], only three major databases (PubMed, Scopus, and Cochrane) were systematically searched. While these are widely used in clinical research, the exclusion of other databases may have limited the comprehensiveness of our search and potentially introduced publication bias [63].

Second, there was substantial technical heterogeneity among the included studies in terms of thermographic devices and imaging protocols. The studies employed a wide range of systems, including FLIR E30 [31], FLIR T650SC [36,37,38,39,40], FLIR E5 [33,34], FLIR T335 [44], IRIS 5000 [43], ThermaCAM SC 500 [30,35], Fluke Ti20 [49], and others. Differences in camera resolution, sensitivity, software, calibration, patient preparation, and imaging environment (e.g., room temperature, humidity, and lighting) were frequently noted [35,36,38,39,40,41,44,47]. Some studies reported detailed imaging protocols [35,36,38,39,40,41,44,47], while others lacked standardization [30,34,41,46,47], which may have affected the comparability and reliability of temperature measurements. Moreover, thermography only measures surface temperature and does not detect deeper ischemic damage [30,52]. Also, other factors may influence skin temperature [32], so the method lacks specificity [31].

Third, the methodological heterogeneity was considerable. The included studies varied in design (case reports, cross-sectional, prospective observational studies, randomized controlled trials), stroke phase (acute vs. chronic), and clinical aims (diagnosis, monitoring, or rehabilitation assessment) [9,30,31,32,33,34,35,36,37,38,39,40,41,43,44,45,46,47,48,49]. For instance, temperature differences ranged widely—from <0.5 °C to >2.5 °C depending on body region and condition assessed [31,34,38,40], and the presence of control groups was inconsistent.

Lastly, the review was limited to English-language full-text publications, potentially introducing language and selection bias [63]. This restriction may have excluded relevant studies published in other languages, and the omission of grey literature or conference proceedings further narrows the scope of included evidence [63].

### 4.7. Future Research

Future research in thermography for stroke should focus on addressing these limitations. Large-scale, multicenter studies with standardized protocols are needed to establish the clinical utility of thermography in stroke diagnosis and management. Research should also explore the potential of combining thermography with other imaging modalities and clinical assessments to improve diagnostic accuracy. Furthermore, studies should investigate the use of artificial intelligence and machine learning algorithms to enhance the interpretation of thermographic data, potentially leading to more accurate and efficient stroke detection and classification. The aim may be to produce a robust protocols similar to the one created for the Wallenberg syndrome [50].

## 5. Conclusions

Thermography shows promise as a non-invasive, radiation-free tool for supporting stroke assessment and monitoring. While current studies suggest associations with autonomic and perfusion-related changes in stroke patients, its clinical utility remains investigational. Further high-quality research is needed to validate its diagnostic and prognostic value, and to determine whether it can meaningfully complement established imaging modalities in improving patient care.

## Figures and Tables

**Table 1:** Summary of the included studies. Abbreviations: BST—Body Surface Temperature, CPSP—Central Post-stroke Pain, DM—Diabetes Mellitus, FLIR—Forward Looking Infrared, LD—Laterality Difference, MPC—Medial Palpebral Commissure, NCV—Noncentral Vertigo, VAS—Visual Analog Scale, VI—Vastus Intermedius, WS—Wallenberg Syndrome, NA—Not Available/Not Applicable.

Author, Year Country Citation	Study Design	Number of Participants	Thermography System; Software	Visualized Area of Body	Intended Use	Results	Conclusions
***ACUTE***							
Korpelainen, 1995 Finland [9]	Prospective follow-up study	63	Digital thermometer (TC-1100, Line Seiki)	Whole body	To evaluate skin temperature asymmetry and its relation to autonomic dysfunction.	There was asymmetry in skin temperatures in patients with brain infarction, with the limbs contralateral to the infarction being markedly colder than the ipsilateral ones.	In hemispheric brain infarction, the coldness of the paretic limbs is associated with clinical signs of the pyramidal tract lesion.
Park, 2022 South Korea [30]	Case report	1	Therma CAMTM SC 500; FLIR tools	Upper and lower limb	To quantitatively evaluate differences in body temperature of limbs and the body to monitor stroke patients’ condition.	Lacunar infarction in the basal ganglia caused an anomaly in the white matter pathway between their patient’s brainstem and cerebral cortex, which caused body temperature to drop opposite to the side of the lesion (~2 °C difference).	People with acute lacunar infarction syndrome may experience issues with thermoregulation, along with cerebral hemisphere and brain stem infarction, and thermography may help with its detection.
Piskorz, 2016 Poland [31]	Cross-sectional study	38	FLIR E 30; FLIR tools	Head	To evaluate temperature differences in the brain as a diagnostic tool for strokes, and differentiate between different types of strokes (ischemic and hemorrhagic).	Temperature decreased in the stroke area compared to the healthy area. The difference was statistically significant (*p* < 0.05) both on the first and fourth day. The average temperature change was 0.49 °C on day 1 and 0.38 °C on day 4. No significant difference was found between ischemic and hemorrhagic stroke.	Thermography is a useful tool for stroke diagnosis, but it does not differentiate between hemorrhagic and ischemic strokes. Temperature change does not depend on the size of the focal area in CT and it does not correlate with the clinical status of patients.
Stokholm, 2021, Denmark [32]	Prospective, longitudinal, observational study	64	FLIR T430sc; FLIR tools	Face	To measure facial temperature in acute stroke patients to investigate its potential as a marker for delirium.	There was no difference between facial temperature measurements while delirium was present and measurements obtained while delirium was absent except in medial palpebral commissure (MPC) region where skin temperature was significantly lower during delirium (difference of 0.40 °C, 95% CI (0.72 to 0.08), *p* value = 0.014).	Since skin is under autonomic control and delirium can cause autonomic dysregulation, they think temperature can show the occurrence of delirium. The only facial region to show signs of delirium is the medial palpebral commissure. Biological sex and body temperature both have “significant association” with facial temperature, so they should be considered when using facial thermography.
Takahashi, 2018 Japan [33]	Observational study	18	FLIR E5; FLIR tools	Whole body	To measure body surface temperature and its laterality in patients with acute Wallenberg’s syndrome (WS) compared to pontine infarction patients, to aid in diagnosis.	A total of 89% of WS patients showed a laterality of body surface temperature (BST) at multiple sites, some with whole-body laterality, in contrast to pontine infarction patients, and the BST laterality decreased over time.	Thermography can help prevent misdiagnosis of acute Wallenberg’s syndrome.
Takahashi, 2024 Japan [34]	Observational study	48	FLIR E5; FLIR tools	Whole body	To measure body surface temperature (BST) to differentiate Wallenberg’s syndrome from noncentral vertigo in the acute phase.	BST findings observed only in patients with WS included (i) LD ≥ 1.0 °C in the abdomen, (ii) LD ≥1.5 °C in the upper limbs, (iii) LD ≥ 2.5 °C, and (iv) LD ≥ 0.5 °C in the four ipsilateral locations. However, the sensitivity in WS was low at (i) 33%, (ii) 22%, (iii) 56%, and (iv) 22%, respectively. None of these findings were observed in patients with NCV.	Thermography may be useful for detecting temperature differences in Wallenberg’s syndrome compared to noncentral vertigo.
***CHRONIC***							
Alfieri, 2016 Brazil [35]	Cross-sectional study	100	ThermaCAM SC 500; FLIR tools	Upper and lower limb	To measure cutaneous temperature in hemiplegic patients, in comparison with healthy subjects, to investigate thermal sensitivity after stroke.	Individuals with stroke sequelae present lower temperature in the paretic side, especially on their feet.	Thermography reveals temperature asymmetry in stroke patients, with the paretic side being cooler.
Alfieri, 2019 Brazil [36]	Case report	1	FLIR T650SC; FLIR Tools	Lower limbs, feet	To evaluate the cutaneous temperature distribution before, during, and after robotic therapy for gait training in a stroke patient.	Temperature values increased immediately after robotic gait training, followed by a thermoregulation 30 min after rest. For example, temperature differences in the thigh region changed from 1.1 °C at baseline to 2.0 °C immediately after the training, then to 0.3 °C after 30 min.	Thermography can track temperature changes in stroke patients undergoing robotic therapy. Difference between the sides normalizes after training.
Alfieri, 2020 Brazil [37]	Case-control, cross sectional observational study	16	FLIR T650SC; FLIR Tools	Plantar region of both feet	To assess plantar cutaneous temperature in patients with stroke, and in those with stroke and diabetes mellitus.	There were no significant differences in temperature between the stroke with DM and stroke only groups.	Diabetes was not shown to cause additional difference in temperature of plegic/non-plegic limb among patient with stroke.
Alfieri, 2023 Brazil [38]	Cross-sectional study	14	FLIR T650SC; FLIR Tools	Whole body	To detect thermal asymmetry in patients with stroke sequelae using a portable, low-cost camera, and to assess its agreement with a high-resolution device.	The FLIR C5 showed adequate-to-excellent general concordance with the FLIR T650sc. Temperature differences were found between the plegic and contralateral side with the values being lower in the plegic side by 0.5 °C in the hand (anterior view), 1.4 °C in the leg (anterior view), 0.8 °C in the hand (posterior view), and 1.5 °C in the leg (posterior view).	A portable thermographic camera can be used reliably to measure thermal asymmetry in stroke patients.
Da Silva Dias, 2021, Brazil [39]	Cross-sectional study	86	FLIR T650SC; FLIR Tools	Upper and lower limb, feet	To quantify skin temperature of each limb to examine the association between temperature, tactile sensibility, and sensorimotor recovery after stroke.	There was an association between temperature differences and reports of sensation. Among those reporting thermal alterations, a higher temperature difference was associated with increased tactile sensibility difference. The opposite was found in those not reporting thermal alterations.	Thermography can help to evaluate the association between temperature differences and tactile sensation in stroke patients
Da Silva Dias, 2022 America [40]	Cross-sectional study	43	FLIR T650SC; FLIR tools	Upper and lower limb	To assess whole-body temperature distribution in stroke patients who report temperature differences between sides of the body.	The plegic limb had significantly lower temperatures than the contralateral side in all segments evaluated. The overall mean temperature difference was 0.7 °C, with the most significant differences in the dorsal forearm (0.8 °C), ventral leg (0.7 °C), and dorsal hand (0.6 °C).	Thermography can detect temperature differences in stroke patients who report feeling cold in their plegic limb.
Gomes, 2022 Brazil [41]	Cross-sectional study	24	FLIR 72,001; FLIR Tools	Face	To record the thermographic patterns of the masseter and temporalis muscles in patients after hemorrhagic stroke, and compare with healthy controls.	No significant differences in skin temperature were found in the masseter and temporal muscles between the post-hemorrhagic stroke and control groups. A significant difference (*p* < 0.05) was noted in the thickness of the left temporal muscle at rest (*p* = 0.01).	Thermography may not detect temperature differences in the masticatory muscles after hemorrhagic stroke, but other changes may be detectable using other methods
Hegedus, 2017 Hungary [42]	Randomized controlled trial	16	Fluke Ti20, Fluke Corporation	Upper limbs	To monitor the effectiveness of stroke rehabilitation treatment by measuring changes in microcirculation and joint function.	Microcirculatory dysfunction was found in all affected extremities. Following treatment, temperature significantly increased (*p* ≥ 0.5 °C) on the affected side. A strong correlation was found between joint function and temperature change (*p* < 0.05).	Thermography is a reliable method for monitoring the effects of stroke rehabilitation treatment.
Kim, 2006 South Korea [43]	Prospective cohort study	70	IRIS 5000, Medicore	Whole body	To correlate temperature changes with pain relief in central poststroke pain as measured by VAS scores and using infrared thermography.	The skin temperature of the pain site was significantly lower than the non-pain site before treatment and improved after treatment, in accordance with improvement of VAS pain scores, Significant correlation between the change of pain and temperature in CPSP (Central post-stroke pain) patients was found.	It is suggested that infrared thermography is very useful device for the evaluation of central post-stroke pain and its treatment.
Nowak, 2020 Poland [44]	Prospective, single-centre study	40	FLIR T335; FLIR tools	Lower limbs	To measure the effects of rehabilitation on spasticity in stroke patients, by assessing the surface temperature of the shank.	The temperature difference between the spastic and non-spastic shank was 0.78 °C at baseline and 0.50 °C after rehabilitation	Thermography can be used to measure the effects of rehabilitation on spasticity in stroke patients.
Sánchez-Sánchez, 2019, Spain [45]	Cross-sectional study	44	FLIR E60bx; FLIR tools	Lower limbs	To assess cutaneous temperature variation as a marker of muscle metabolism in stroke patients.	A temperature difference of −0.57 °C was found between the paretic and non-paretic thigh in the group with limited ambulation.	Paretic VI (vastus intermedius) muscle wasting may be an important factor to reach normal walking.
Satoh, 2002 Japan [46]	Case report	1	NA	Upper limbs	To monitor skin temperature decrease and somatosensory function disturbances following stroke.	Ischemic stroke of the postcentral gyrus was associated with somatosensory and skin temperature disturbances. After cooling, the affected hand still had a low skin temperature; it was lower on the ulnar side than on the radial side, while the skin temperature had recovered completely on the unaffected hand.	Thermography can help detect sensory disturbances after stroke.
Wanklyn, 1994 United Kingdom [47]	Observational study	21	Starsight Thermographic camera; Insight Vision Systems	Upper limbs	To objectively verify temperature differences between hemiplegic and normal arms.	During cold stress, the median temperature difference between non-hemiplegic and hemiplegic hands was 0.65 °C at pre-cooling, 0.1 °C at 0 min, 2.035 °C at 3 min, 0.47 °C at 5 min, and 0.65 °C at 10 min, and for patients not reporting cold, these values were −0.2 °C, 0 °C, 0 °C, −0.72 °C, and −1.4 °C respectively.	Thermography is useful for assessing the cold hemiplegic arm in stroke.
Zanona, 2018 Brazil [48]	Single group pre/post intervention study	10	C2 Camera, Flir Tools	Upper and lower limb	To analyze body asymmetry as an indicator of the effects of virtual reality rehabilitation on stroke patients.	Use of virtual reality-based rehabilitation resulted in improved symmetry of body temperature, with changes including the right forearm (+1.23 °C), previous direct hand (+1.56 °C), rear right hand (+1.28 °C), and left back hand (+0.9 °C).	VR can improve body temperature symmetry after stroke, as measured by thermography.

## Data Availability

Data available at OSF, https://doi.org/10.17605/OSF.IO/6S3DU.

## Supplementary Materials

The following supporting information can be downloaded at: https://www.mdpi.com/article/10.3390/medicina61050854/s1, Table S1. Details on reason of full text exclusion with additional reference list; Table S2. Details of the included studies on thermography imaging. Table S3. Detailed assessment of the risk of bias.

## Abbreviations

The following abbreviations are used in this manuscript:

PRISMA	Preferred Reporting Items for Systematic Reviews and Meta-Analyses
ROBINS-I	Risk Of Bias In Non-Randomized Studies—of Interventions
CVA	Cerebrovascular Accident
BST	Body Surface Temperature
LD	Laterality Difference
VAS	Visual Analog Scale
NCV	Noncentral Vertigo
IRIS	Infrared Imaging System
AI	Artificial Intelligence
MRI	Magnetic Resonance Imaging
CT	Computed Tomography
