# Supplementary material for: Thermography in Stroke—A Systematic Review
Source: Medicina (Kaunas). 2025 May 6;61(5):854. doi: 10.3390/medicina61050854 (PMC12113360; doi:10.3390/medicina61050854)
Supplement: Supplementary file 1 [file medicina-61-00854-s001.zip › medicina-3534889-supplementary.pdf]

medicina MDPI

Supplementary Table S1. Details on reason of full text exclusion with additional reference list

**Reason for exclusion**	**Full text**
Non-human	1–3
Non-infrared thermography	4–7
Non-stroke	8–16
Systematic review or meta-analysis	17–19

1. Mityagin KS, Zaretskiy AP, Prokhorov IB. Simulation for dynamics of transient ischemic attacks with thermal infrared imaging. In: IOP Conf. Ser. Mater. Sci. Eng. Institute of Physics Publishing. Epub ahead of print 2018. DOI: 10.1088/1757-899X/450/4/042012.
2. Suzuki T, Oishi N, Fukuyama H. Simultaneous infrared thermal imaging and laser speckle imaging of brain temperature and cerebral blood flow in rats. J Biomed Opt; 24. Epub ahead of print 2019. DOI: 10.1117/1.JBO.24.3.031014.
3. Dehkharghani S, Fleischer CC, Qiu D, et al. Cerebral temperature dysregulation: MR thermographic monitoring in a nonhuman primate study of acute ischemic stroke. Am J Neuroradiol 2017; 38: 712–720.
4. Friedlander AH, Gratt BM. Panoramic dental radiography as an aid in detecting patients at risk for stroke. J Oral Maxillofac Surg 1994; 52: 1257–1262.
5. Karaszewski B, Carpenter TK, Thomas RGR, et al. Relationships between brain and body temperature, clinical and imaging outcomes after ischemic stroke. J Cereb Blood Flow Metab 2013; 33: 1083–1089.
6. Management of carotid atherosclerosis in stroke - PubMed, https://pubmed.ncbi.nlm.nih.gov/38589215/ (accessed 11 April 2025).
7. Riedl B, Beckmann T, Neundörfer B, et al. Autonomic failure after stroke--is it indicative for pathophysiology of complex regional pain syndrome? Acta Neurol Scand 2001; 103: 27–34.
8. Alfieri FM, Battistella LR. Body temperature of healthy men evaluated by thermography: A study of reproducibility. Technol Health Care 2018; 26: 559–564.

9. Cabizosu A, Grotto D, López López A, et al. Thermography Sensor to Assess Motor and Sensitive Neuromuscular Sequels of Brain Damage. Sensors; 24. Epub ahead of print 2024. DOI: 10.3390/s24061723.
10. Capistrant TD, Gumnit RJ. Thermography and extracranial cerebrovascular disease. Preliminary report of a new provocative technique. Arch Neurol 1970; 22: 499–503.
11. Gorbach AM, Heiss J, Kufta C, et al. Intraoperative infrared functional imaging of human brain. Ann Neurol 2003; 54: 297–309.
12. Lin J, Wu Y, Deng X, et al. Application of intraoperative infrared thermography in bypass surgery for adult moyamoya syndrome: A preliminary study. Front Neurol; 14. Epub ahead of print 2023. DOI: 10.3389/fneur.2023.1174072.
13. Price TR, Heck AF. Correlation of Thermometry and Angiography in Carotid Arterial Disease: Thermometry as a Screening Technique. Arch Neurol 1972; 26: 450–455.
14. Toutouzas K, Benetos G, Drakopoulou M, et al. Incremental predictive value of carotid inflammation in acute ischemic stroke. Stroke 2015; 46: 272–274.
15. Toutouzas K, Benetos G, Drakopoulou M, et al. Insights from a thermography-based method suggesting higher carotid inflammation in patients with diabetes mellitus and coronary artery disease. Diabetes Metab 2014; 40: 431–438.
16. Watson JC, Gorbach AM, Pluta RM, et al. Real-time detection of vascular occlusion and reperfusion of the brain during surgery by using infrared imaging. J Neurosurg 2002; 96: 918–923.
17. Neves EB, Vilaça-Alves J, Rosa C, et al. Thermography in neurologic practice. Open Neurol J 2015; 9: 24–27.
18. Grotto D, Luceño-Sanchez JA, Cabizosu A. Thermography in the analysis of physiotherapeutic treatment in patients with brain damage: A systematic review. Cuestiones de Fisioterapia 2023; 52: 35–50.
19. Mikulska D. [Contemporary applications of infrared imaging in medical diagnostics]. Ann Acad Med Stetin 2006; 52: 35–39; discussion 39-40.

Supplementary Table S2. Details of the included studies on thermography imaging. Abbreviations: BST – Body Surface Temperature, IL – Inferior Labial, LC – Labial Commissure, LPC – Lateral Palpebral Commissure, MPC – Medial Palpebral Commissure, NL – Nasolabial, ROI – Region of Interest, ST – Supratrochlear, TEMP – Temporal, VAS – Visual Analog Scale, VI – Vastus Intermedius, NA – Not Available / Not Applicable.

Author, Year<br>Country<br>Citation	Instructions prior to thermographic imaging	Room specific	Body position	Region of interest	Camera position / distance
ACUTE					
Korpelainen, 1995<br>Finland<br>[9]	- Patients were acclimatized to room temperature for 30 minutes<br>- Patients were asked about the presence of a feeling of cold on either side of the body	- Room temperature was 24.0 ± 0.5°C<br>- Humidity 47%<br>- Draft free	Reclining position	NA	NA
Park, 2022<br>South Korea<br>[30]	NA	NA	NA	NA	NA
Piskorz, 2016<br>Poland<br>[31]	Patients' heads weren't cooled for two hours before the tests to help get reliable temperature results	NA	NA	Temporal area – bilaterally	NA
Stokholm, 2021,<br>Denmark<br>[32]	NA	No air conditioning to keep temperature within a narrow range; ambient temperatures ranged from 20.7°C to 28.9°C	Frontal view	Seven ROIs on each side of the face: Supratrochlear (ST), Temporal (TEMP), Lateral palpebral commissure (LPC), Medial palpebral commissure (MPC), Nasolabial (NL), Labial commissure (LC) and Inferior labial (IL)	Varied to fill frame
Takahashi, 2018<br>Japan<br>[33]	NA	NA	NA	Nasolabial fold on the face., Palm of the hand., Thoracic spine level 8-10 area (approx. 5cm from the umbilicus)., Centre of the dorsum of the foot.	NA
Takahashi, 2024<br>Japan<br>[34]	NA	Unregulated	Sitting or supine position, depending on what's comfortable for their dizziness.	Face, upper limbs, abdomen, lower limbs	NA
CHRONIC					
Alfieri, 2016<br>Brazil<br>[35]	◦ No hot showers / baths<br>- No lotion or talcum powder<br>- No heavy exercise	- Temperature was 22◦C<br>- Windows + curtains were closed<br>- Cold fluorescent lights	NA	Hand, feet, axillary temperature	NA

	- No heavy meals<br>- No drinking stimulants<br>- No medication<br>- Remove gloves + socks / shoes				
Alfieri, 2019<br>Brazil<br>[36]	- No shower/bath<br>- No ointments/body powder<br>- No vigorous exercises<br>- No scratching<br>- 15 min in climatized room at 21.2∘ C	- Doors, windows, + curtains closed<br>- Humidity 64%.<br>- Cold fluorescent lights		Thigh: 5cm above the patella upper border and the inguinal line. Leg: 5cm below the patella lower border and 10cm above the malleolus. Other ROIs: calcaneus, surface of the fifth, third, and first toes, and the medial and lateral regions of the feet.	1.5-4 meter
Alfieri, 2020<br>Brazil<br>[37]	NA	NA	- 1.5m from camera (ref 12) for calcaneus, the surface of the 1st, 3rd, 5th toes with corresponding metatarsal regions; patient was in supine position with slight dorsiflexion	Plantar region including the calcaneus, hallux, third and fifth toes, and their respective metatarsal regions.	1.5 meter
Alfieri, 2023 Brazil<br>[38]	- No hot showers / baths<br>- No spreading creams or powders<br>- No exercise 2 hours before images<br>- No drinking stimulants<br>- No nasal decongestants<br>- No smoking	Thermal laboratory, room had varying temperatures	- Both cameras were perpendicular to patients<br>- Patients were in orthostatic position	Whole body	- 4m and 1.5m from camera<br>- 0.4m from the wall
Da Silva Dias, 2021, Brazil<br>[39]	- No hot showers / baths<br>- No ointments or powder<br>- No heavy exercise 2 hours before session<br>- No eating 2 hours before session<br>- No stimulants<br>- No alcohol or coffee<br>- No smoking<br>- No nasal decongestant	- Temperature was 21∘C<br>- Windows, shutters + doors were closed<br>- 65% humidity	Patients were in orthostatic position for the first test, lying down in the second one	Hand: Junction of the third metacarpal with the third proximal phalanx and the cubital styloid process. Forearm: Distal forearm and cubital fossa. Arm: Cubital fossa and axillar line. Thigh: 5 cm above the upper bound of the patella and the inguinal line. Leg: 5 cm below the lower bound of the patella and 10 cm above the malleolus. Cutaneous region of the feet was also considered.	1.5 meters

Da Silva Dias, 2022 America [40]	- Patients had to wear swimsuits or underwear to uncover limbs<br>- No wearing bracelets, necklaces or watches<br>- No moving arms or legs during assessment<br>- No scratching body before or during assessment	- Average room temperature was 21ºC<br>- Average humidity was 65%<br>- Windows + curtains were closed	Anatomical	Hand: Junction of the third metacarpal with the third proximal phalanx and the cubital styloid process. Forearm: Distal forearm and cubital fossa. Arm: Cubital fossa and axillar line. Thigh: 5 cm above the upper bond of the patella and the inguinal line. Leg: 5 cm below the lower bond of the patella and 10 cm above the malleolus.	- Patients had to stand 4 meters from the infrared sensor<br>- They also had to stand 0.4m away from the wall
Gomes, 2022 Brazil [41]	- No food, analgesies, caffeine or thermogenics for 2 hours before study<br>- No makeup, sunscreen or cream<br>- No pressing or scratching face	- Reduced light<br>- Temperature was 71.6 - 73.4◦F between studies	- Patients were sitting in an upright posture<br>- Patients were 1m from the camera<br>- 90° angle between patients and camera	Masseter and temporalis muscles.	NA
Hegedus, 2017 Hungary [42]	- Patients had to lie down for 15 minutes before the study<br>- No alcohol<br>- No coffee<br>- No cigarettes	- Temperature was 21°C - 23°C between studies<br>- Humidity was 70% - 80%<br>- Draft-free	NA	NA	NA
Kim, 2006 South Korea [43]	-Thermographic patients VAS Scores (0-10) were measured 4 weeks before pain treatment.<br>-Thermographic patients VAS Scores were measured (0-10) again after pain treatment.	- Temperature was 19°C - 20°C<br>- Draft-free	ROI: pain sites and non-pain sites	NA	NA
Nowak, 2020 Poland [44]	- No physical treatments before examination<br>- No exercise<br>- No stimulants<br>- No large meals<br>- Avoid sun exposure<br>- No showering before examination	- Room was 20 m2<br>- Temperature was 22–23 ° C<br>- Humidity was 55%<br>- No radiators or rapid airflow	- Patients were lying in supine position<br>- Plantar-flexed ankles<br>- Hips in neutral rotation and flexion	Posterior part of the shank, designated by a rectangle created by two lines: one passing through the narrowest point between the medial and lateral malleolus and the second passing 10cm proximally.	NA

Sánchez-Sánchez, 2019, Spain [45]	-Patients were asked to remove clothing from legs<br>-Patients were given 10 minutes to acclimatize to their ambient temperature	- Temperature was between 22–23ºC<br>- Humidity 40%	- Stand still with legs separated	Quadriceps muscle (thigh). Also includes assessment of bilateral lower limbs	1 m- Camera set vertically at the center point between both thighs, at a distance of 1m.
Satoh, 2002 Japan [46]	- Thermography done on hands in a resting state<br>- Thermography was performed a second time after the hands were placed in ice water for 15 seconds, followed by 15 minutes of recovery.	NA	NA	NA	NA
Wanklyn, 1994 United Kingdom [47]	-questionnaire asked feeling of: coldness, heat, pain, pins and needles in the arm<br>-patients were acclimatized to room temperatures for 30 minutes<br>- and then seated in a constant temperature of 25 ± 1°C<br>-Latex gloves were placed on the hands and immersed in water at 5°C for 1 minute<br>- patients were asked to keep the hands still and not allow them to touch the sides of the container.	25 ± 1°C	Sitting	NA	NA
Zanona, 2018 Brasil [48]	- no vigorous exercises in the last 24 h<br>- no alcohol / caffeine<br>- no cream or lotion in the last 6 h<br>- no scratching for last 10 min	- Temperature between 23 and 24°C<br>- humidity 42-50%<br>- fluorescent light	Standing	Hand: Junction of the 3rd proximal phalanx of the metacarpal with the 3rd styloid ulnar process. Forearm: Cubital fossa up to distal forearm. Arm: Cubital fossa up to axillary line. Thigh: 5 cm above the upper limit of the patella and inguinal line. Leg: 5 cm below the patella and 10 cm above the malleolus.	1.5 m

Supplementary Table S3. Detailed assessment of the risk of bias.

**Author, Year Country Citation**	**Risk of bias tool**	**Detailed Assessment**	**Overall risk of bias**
Korpelainen, 1995 [9]	Newcastle-Ottawa Scale (NOS)	Selection: Good; Comparability: Fair; Outcome: Good	Moderate
Park, 2022 [30]	JBI Checklist for Case Reports	Good	Low
Piskorz, 2016 [31]	QUADAS-2	Patient Selection: Low; Index Test: Low; Reference Standard: Unclear; Flow/Timing: Low	Moderate
Stokholm, 2021 [32]	Newcastle-Ottawa Scale (NOS)	Selection: Good; Comparability: Good; Outcome: Good	Low
Takahashi, 2018 [33]	ROBINS-I	Confounding: Low; Selection Bias: Low; Measurement: Moderate; Missing Data: Low	Low
Takahashi, 2024 [34]	ROBINS-I	Confounding: Low; Selection Bias: Low; Measurement: Moderate; Missing Data: Low	Low
Alfieri, 2016 [35]	AXIS tool	Reporting: Moderate; Methods: Good; Bias: Low	Moderate

Alfieri, 2019 [36]	JBI Checklist for Case Reports	Good	Low
Alfieri, 2020 [37]	JBI Checklist for Case-Control Studies	Selection: Fair; Comparability: Fair; Outcome: Good	Moderate
Alfieri, 2023 [38]	AXIS tool	Reporting: Moderate; Sample: Moderate; Methods: Good	Moderate
Da Silva Dias, 2021 [39]	AXIS tool	Reporting: Moderate; Methods: Moderate; Bias: Low	Moderate
Da Silva Dias, 2022 [40]	AXIS tool	Reporting: Moderate; Methods: Moderate; Bias: Low	Moderate
Gomes, 2022 [41]	AXIS tool	Reporting: Moderate; Methods: Moderate; Bias: Moderate	Moderate
Hegedus, 2017 [42]	RoB 2	Randomization: Good; Deviation: Fair; Missing Outcome: Fair; Measurement: Good; Selection: Good	Moderate
Kim, 2006 [43]	Newcastle-Ottawa Scale (NOS)	Selection: Good; Comparability: Good; Outcome: Good	Low

Nowak, 2020 [44]	Newcastle-Ottawa Scale (NOS)	Selection: Good; Comparability: Good; Outcome: Good	Low
Sánchez-Sánchez, 2019, [45]	AXIS tool	Reporting: Moderate; Sample: Fair; Bias: Low	Moderate
Satoh, 2002 [46]	JBI Checklist for Case Reports	Good	Low
Wanklyn, 1994 [47]	Newcastle-Ottawa Scale (NOS)	Selection: Fair; Comparability: Fair; Outcome: Good	Moderate
Zanona, 2018 [48]	ROBINS-I	Confounding: Moderate; Selection bias: Moderate; Measurement: Low; Missing data: Moderate; Reporting bias: Moderate	Moderate
